# Mechanistic Effects of Baicalein on Aqueous Humor Drainage and Intraocular Pressure

**DOI:** 10.3390/ijms23137372

**Published:** 2022-07-01

**Authors:** Hoi-lam Li, Sze Wan Shan, W. Daniel Stamer, King-kit Li, Henry Ho-lung Chan, Mortimer M. Civan, Chi-ho To, Thomas Chuen Lam, Chi-wai Do

**Affiliations:** 1 School of Optometry, The Hong Kong Polytechnic University, Hong Kong; hoilam.k.li@gmail.com (H.-l.L.); samantha.shan@polyu.edu.hk (S.W.S.); kk.li@polyu.edu.hk (K.-k.L.); henryhl.chan@polyu.edu.hk (H.H.-l.C.); chi-ho.to@polyu.edu.hk (C.-h.T.); thomas.c.lam@polyu.edu.hk (T.C.L.); 2 Centre for Eye and Vision Research (CEVR), 17W Hong Kong Science Park, Hong Kong; 3 Research Centre for SHARP Vision (RCSV), The Hong Kong Polytechnic University, Hong Kong; 4 Research Centre for Chinese Medicine Innovation (RCMI), The Hong Kong Polytechnic University, Hong Kong; 5 Department of Ophthalmology, Duke University, Durham, NC 27708, USA; william.stamer@duke.edu; 6 Department of Biomedical Engineering, Duke University, Durham, NC 27708, USA; 7 Department of Physiology, University of Pennsylvania, Philadelphia, PA 19104, USA; civan@mail.med.upenn.edu; 8 Research Institute of Smart Ageing (RISA), The Hong Kong Polytechnic University, Hong Kong

**Keywords:** Baicalein, intraocular pressure, outflow facility, trabecular meshwork, cell contractility, regulatory volume decrease, extracellular matrix, proteomics, glaucoma

## Abstract

Elevated intraocular pressure (IOP) is a major risk factor for glaucoma that results from impeded fluid drainage. The increase in outflow resistance is caused by trabecular meshwork (TM) cell dysfunction and excessive extracellular matrix (ECM) deposition. Baicalein (Ba) is a natural flavonoid and has been shown to regulate cell contraction, fluid secretion, and ECM remodeling in various cell types, suggesting the potential significance of regulating outflow resistance and IOP. We demonstrated that Ba significantly lowered the IOP by about 5 mmHg in living mice. Consistent with that, Ba increased the outflow facility by up to 90% in enucleated mouse eyes. The effects of Ba on cell volume regulation and contractility were examined in primary human TM (hTM) cells. We found that Ba (1–100 µM) had no effect on cell volume under iso-osmotic conditions but inhibited the regulatory volume decrease (RVD) by up to 70% under hypotonic challenge. In addition, Ba relaxed hTM cells via reduced myosin light chain (MLC) phosphorylation. Using iTRAQ-based quantitative proteomics, 47 proteins were significantly regulated in hTM cells after a 3-h Ba treatment. Ba significantly increased the expression of cathepsin B by 1.51-fold and downregulated the expression of D-dopachrome decarboxylase and pre-B-cell leukemia transcription factor-interacting protein 1 with a fold-change of 0.58 and 0.40, respectively. We suggest that a Ba-mediated increase in outflow facility is triggered by cell relaxation via MLC phosphorylation along with inhibiting RVD in hTM cells. The Ba-mediated changes in protein expression support the notion of altered ECM homeostasis, potentially contributing to a reduction of outflow resistance and thereby IOP.

## 1. Introduction

Glaucoma is an incurable eye disorder characterized by the loss of retinal ganglion cells (RGCs), leading to permanent blindness. The number of glaucoma patients is expected to exceed 110 million globally by 2040 [1]. Primary open-angle glaucoma (POAG) is the most common form with insidious onset and progression. Elevated intraocular pressure (IOP), as determined by the balance between aqueous humor (AH) secretion and drainage rates, is widely recognized as an important risk factor. Normally, AH nourishes avascular ocular tissues and carries metabolic waste and debris out of the eyes [2]. However, in glaucoma, elevated IOP due to restricted AH drainage damages the eyesight. As such, IOP-lowering interventions remain the only effective clinical treatment to retard glaucomatous vision loss [3]. However, prolonged use of anti-glaucoma medications often leads to undesirable side effects and drug resistance, significantly affecting patient compliance and treatment outcomes. Considering the rapidly growing elderly population, effective well-tolerated treatment for glaucoma is urgently needed.

In POAG, elevated IOP is primarily mediated by increased resistance to AH drainage [4]. There are two major drainage routes by which AH leaves the eye: the conventional and unconventional pathways [5]. The conventional outflow route accounts for about 80% of AH drainage and its resistance occurs primarily at the junction between the outermost portion of trabecular meshwork (TM) and the inner wall of Schlemm’s canal [6]. The precise mechanisms by which conventional outflow is controlled are not entirely clear, but its resistance can be reduced by several inter-dependent mechanisms including (1) reducing the TM cell volume; (2) relaxing the TM cells; and (3) increasing the degradation of extracellular matrix (ECM) [7]. The amounts and types of ECM, which is composed of several proteins including fibronectin, elastin, and collagens, present in the region can be modulated by matrix metalloproteinases (MMPs) [8,9]. Loss of TM cells may also contribute to increased outflow resistance because of the reduced degradation of ECM materials [10].

Baicalein (Ba) is a single compound extracted from the roots of Scutellaria baicalensis Georgi. It is a natural flavonoid and has potent anti-inflammatory, anti-oxidative, and anti-apoptotic properties [11]. Because of that, Ba has been used in the clinical treatment of cancers, inflammation, and cardiovascular diseases as it was considered to be safe [11,12,13]. Ba has also been proposed to have therapeutic potential in the treatment of ocular diseases [14,15]. As Ba was demonstrated to regulate fluid secretion, cell contraction, and ECM homeostasis in various cell types [11,16,17], raising the possibility that it may regulate outflow resistance and IOP. In this study, we determined whether Ba produced detectable changes in IOP and conventional outflow facilities using in vivo and ex vivo mouse eyes. For in vitro studies, we evaluated the effects of Ba on volume regulation and contractile properties of primary human TM (hTM) cells. In consideration that Ba may act on multiple pathways, a comprehensive exploration of the mechanisms underlying its regulation of the outflow facility was warranted. This was achieved by elucidating the protein expression profile and signaling cascades in Ba-treated hTM cells using an isobaric tag for relative and absolute quantitation (iTRAQ)-based quantitative proteomics.

## 2. Results

### 2.1. Ba Lowers IOP in Mouse Eyes

**Figure 1:**
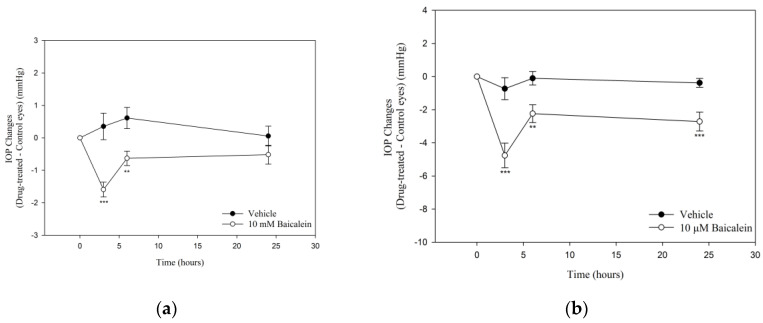
Effects of Baicalein (Ba) on IOP in C57BL/6J mice. (**a**) Ba (10 mM) or vehicle was applied topically to the treatment eye, while the contralateral eye was left untreated as control (*n* = 9 in each group); and (**b**) Ba (2 µL, 10 µM) or vehicle was applied intravitreally to the treatment eye, while the contralateral eye was left untreated as control. Results were expressed as mean ± standard error of the mean (SEM). (*n* = 7–9, ** *p* < 0.01, *** *p* < 0.001, one-way repeated measures ANOVA).

To determine the ocular hypotensive effects, IOP was measured after topical and intravitreal administration of Ba in C57BL/6J mice (Figure 1). A significant IOP reduction was observed after topical application of Ba for 3- and 6-h. The maximum IOP-lowering effect was found to be 1.6 ± 0.2 mmHg (*p* < 0.001) at 3 h after Ba treatment (Figure 1a). No ocular hypotensive effect was observed 24 h after Ba treatment. In addition, the effect of intravitreal administration of Ba was studied. Our results showed that Ba produced a sustained IOP-lowering effect lasting for at least 24 h (Figure 1b). The maximum IOP reduction was found to be 4.8 ± 0.7 mmHg (*p* < 0.001) after 3 h Ba treatment.

### 2.2. Ba Increases Conventional Outflow Facility in Ex Vivo Mouse Eyes

**Figure 2:**
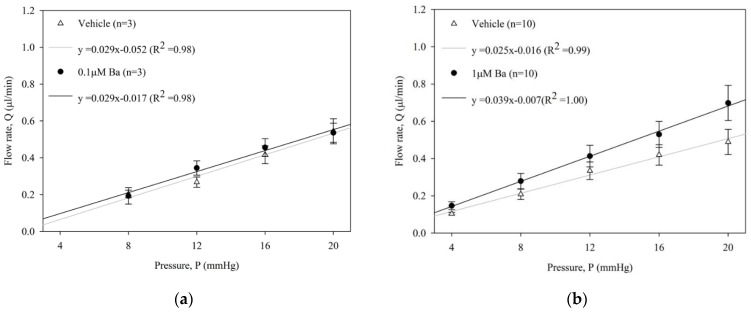

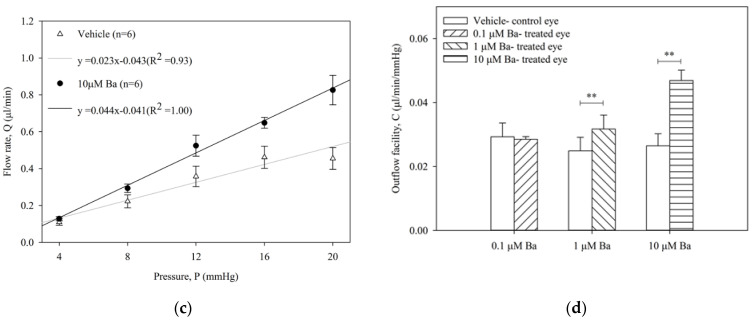
Ba increased outflow facility in ex vivo mouse eyes. Flow rate (Q) measured at different pressures (P) in paired enucleated eyes in C57BL/6J mice with different concentrations of Ba (**a**) 0.1 μM; (**b**) 1 μM; and (**c**) 10 μM. (**d**) The calculated outflow facility of Ba-treated and their contralateral vehicle-treated eyes. (** *p* < 0.01, paired *t*-test).

The effect of Ba (0.1, 1, 10 µM) on outflow facility C (µL/min/mmHg) in enucleated mouse eyes was measured by a constant-pressure perfusion system. The average flow rates of both Ba-treated and vehicle-treated control eyes at each pressure step are presented in Figure 2. No significant difference of outflow facility was shown in control eyes among different groups (range: 0.023 to 0.029 µL/min/mmHg, *p* > 0.05, one-way ANOVA). Our results showed that Ba elicited a concentration-dependent increase in outflow facility. At 0.1 µM, Ba had no effect on outflow facility when compared to the vehicle-treated control eye. At 1 µM and 10 µM, Ba significantly increased outflow facility by 37 ± 13% and 89 ± 20%, respectively.

### 2.3. Mechanistic Studies of Ba in hTM Cells

**Figure A1:**
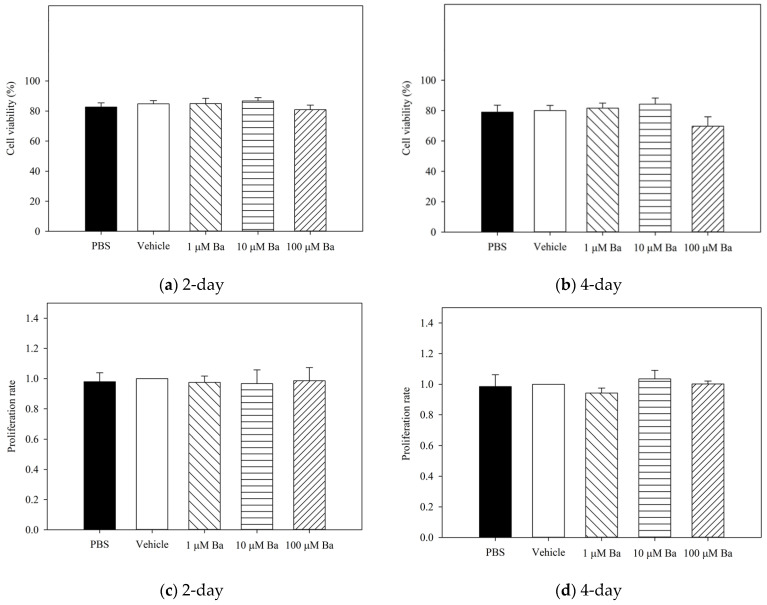
Measurements of hTM cell viability (**a**) and (**b**), and cell proliferation (**c**) and (**d**), normalized with vehicle) after treatment with PBS, vehicle, and Ba for 2 and 4 days. No significant difference in cell viability and proliferation rate were observed among PBS, vehicle- and Ba-treated groups, at both time points. (*n* = 4, *p* > 0.05, one-way ANOVA).

From the preceding in vivo and ex vivo studies, Ba was shown to reduce IOP and outflow resistance. This could be mediated by the modulation of TM cell functions. Before investigating the effects of Ba on cell volume regulation and contractility of hTM cells, we determined the effects of Ba on hTM cell survival and normal cell growth. Our results showed that Ba did not affect cell viability and proliferation (Figure A1), indicating that Ba has low toxicity and a viable safety profile as a pharmacologic agent for subsequent experiments.

#### 2.3.1. Ba Inhibits Cell Volume Regulation in hTM Cells

**Figure 3:**
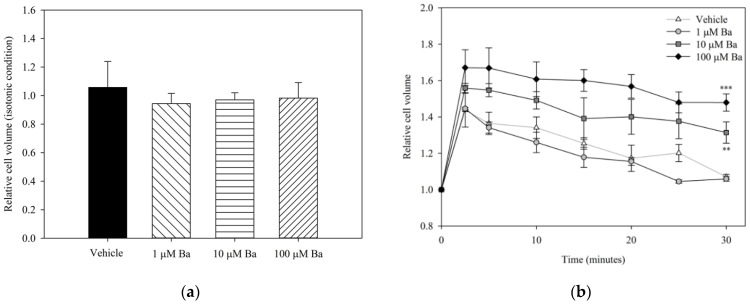
Relative hTM cell volume after treatment with Ba (1, 10, and 100 µM) and vehicle in (**a**) isotonic condition normalized with PBS; and (**b**) upon hypotonic stimulation. (*n* = 3, ** *p* < 0.01, *** *p* < 0.001, one-way ANOVA followed by Bonferroni *t*-test).

To examine the acute effects of Ba on volume regulation, hTM cells were treated with Ba or vehicle under both isotonic and hypotonic conditions. The relative cell volume changes after different treatments are presented in Figure 3. No significant difference was found between vehicle- and Ba-treated groups at concentrations of 1–100 µM under iso-osmotic conditions. The changes in cell volume upon hypotonic stimulation are summarized in Figure 3b. Reducing the osmolarity of the bathing solution triggered a rapid cell swelling followed by a regulatory volume decrease (RVD), that is, a gradual restoration to the initial cell volume. Our results showed that 10 µM and 100 µM Ba significantly inhibited RVD, while 1 µM Ba had no significant effect on cell volume recovery. 

#### 2.3.2. Ba Triggers hTM Cell Relaxation via Inhibition of MLC Phosphorylation

**Figure 4:**
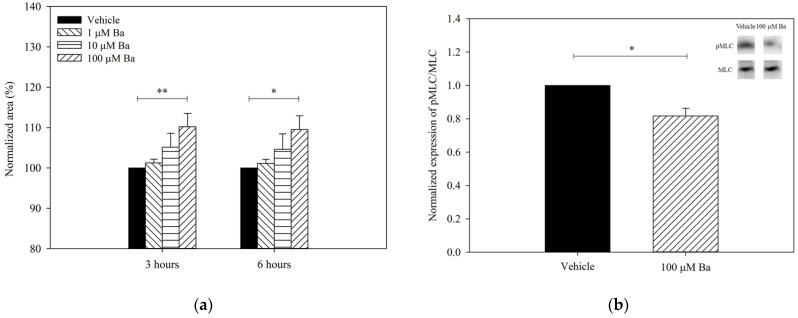
Effects of Ba on the contractility of hTM cells. (**a**) Normalized collagen gel area after treatment with vehicle (*n =* 5), 1 µM (*n =* 3), 10 µM (*n =* 5), and 100 µM (*n =* 5) Ba for 3 and 6 h (* *p* < 0.05, ** *p* < 0.01, two-way repeated measures ANOVA followed by Bonferroni *t*-test) and; (**b**) Normalized expression of pMLC/MLC after vehicle and 100 µM Ba treatment for 5 min using Western blot analyses. (*n =* 4, * *p* < 0.05, paired *t*-test).

To determine with Ba regulates cell contractility, the collagen gels were pre-contracted after seeding with hTM cells, as previously described [18]. The changes in the size of collagen gel containing hTM cells after Ba treatment are summarized in Figure 4a. Ba elicited a concentration-dependent relaxation of hTM cells (increase in gel size). At 100 µM, Ba triggered a sustained relaxation of hTM cells by 10 ± 3% (*p* < 0.01) and 10 ± 3% (*p* < 0.05) after 3- and 6-h treatment, respectively. Carbachol (5 µM) was used as a control. Consistent with previous findings [19], carbachol was shown to trigger a sustained contraction of hTM cells by 14 ± 8% and 13 ± 8% after 3-h and 6-h of treatment, respectively, when compared with the control (*n* = 5, *p* < 0.05). Additionally, the contractility of hTM cells was determined as a percentage of phosphorylated 20-kDa myosin light chain (pMLC) to the total MLC. It was previously demonstrated that there was a positive correlation between cell contraction and the magnitude of pMLC/MLC level [20]. Figure 4b depicts the expression of pMLC/MLC after Ba (100 µM) or vehicle treatment using Western blot analyses. Ba was found to downregulate the expression of pMLC/MLC by 18 ± 9% (*p* < 0.05, paired *t*-test). As expected, carbachol significantly increased the pMLC/MLC expression (*n =* 4, *p* < 0.05, paired *t*-test). Consistent with the findings of the gel contraction assay, our results suggest that Ba triggers the relaxation of hTM cells.

### 2.4. Proteomic Study of hTM Cells following Ba Treatment 

**Figure 5:**
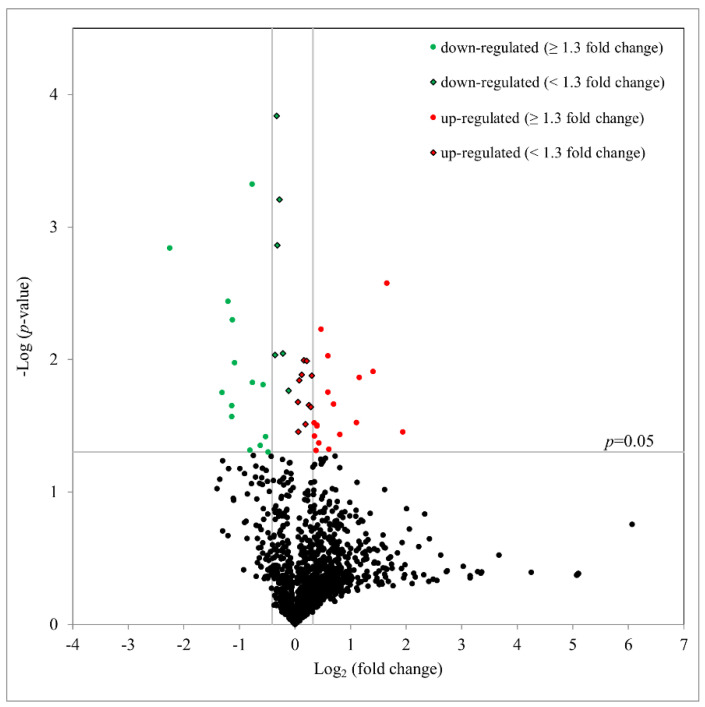
Volcano plot based on proteomics data after Ba (10 µM) treatment for 3 h. The distribution of all proteins with at least two unique peptides is presented. The x-axis shows a log_2_ fold change and y-axis shows the negative log_10_ *p*-value calculated by paired *t*-test. 47 proteins were significantly altered in expression after Ba treatment (*n* = 3, *p* < 0.05). Among those proteins, 17 were upregulated (red circle) and 14 were downregulated (green circle) with at least a 1.3-fold change.

To better understand the underlying mechanisms involved, the protein expression profiles were determined after Ba treatment (10 µM) for 3 h. Using an iTRAQ proteomics, with a false discovery rate (FDR) set to 1%, 1,342 unique hTM cell proteins with at least two unique peptides per protein were identified and quantified. As summarized as a volcano plot in Figure 5, 47 proteins with significantly altered expression (with 27 upregulated and 20 downregulated) after Ba treatment were observed (*p* < 0.05, paired *t*-test). With a fold change of ≥1.3 (log_2_ ≥ 0.31 or log_2_ < −0.42), 17 proteins were upregulated and 14 downregulated after a 3-h treatment (Table 1). In agreement with our foregoing results of Ba-induced functional changes, Ba was shown to upregulate the expression of cathepsin B (CTSB; P07858; *p* < 0.01) and downregulate D-dopachrome decarboxylase (DDT; P30046; *p* < 0.001) and pre-B-cell leukemia transcription factor-interacting protein 1 expression (PBXIP1; Q96AQ6; *p* < 0.05). Additionally, matrix metalloproteinase-14 (MMP-14) was found to be increased by about 1.15-fold (*p* < 0.05).

**Figure 6:**
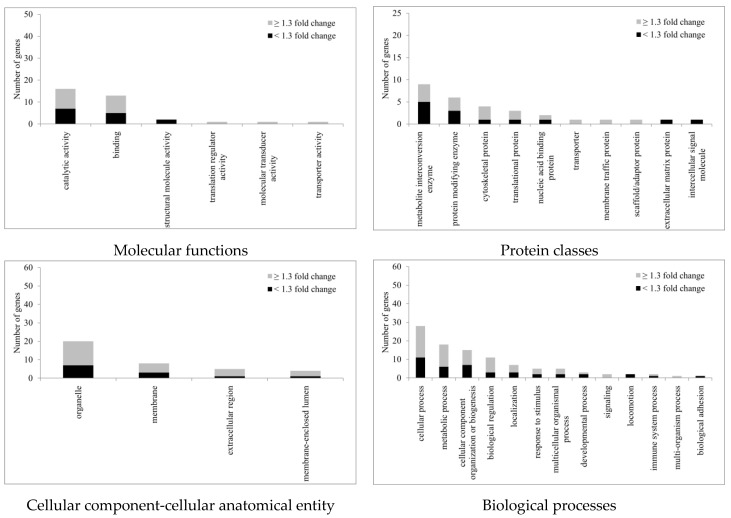
This PANTHER classification with GO annotation of proteins with altered expression after 3 h treatment with Ba (10 µM). (*n* = 3, *p* < 0.05, paired *t*-test).

All statistically significant regulated proteins, regardless of fold changes, were subject to gene ontology (GO) (Homo sapiens) analysis according to the PANTHER classification system (http://www.pantherdb.org, accessed on 3 June 2022). The genes were classified into four categories: molecular functions, protein classes, cellular components, and biological processes (Figure 6). For molecular functions, GO: 0003824 (catalytic activity) was the top regulated one with a total of 16 genes identified. The second ranked GO molecular function was binding (GO: 0005488). Of the protein classes, metabolite inter-conversion enzyme (PC00262) was the top regulated protein class with nine genes identified after Ba treatment. Likewise, for proteins related to the cellular component-cellular anatomical entity, 20 genes were identified with organelle (GO: 0043226) ranked top, followed by membrane (GO: 0016020) with eight genes identified after Ba treatment. For biological processes, the top three were cellular process (GO: 0009987), metabolic process (GO: 0008152), and cellular component organization or biogenesis (GO: 0071840) with 28, 18, and 15 genes, respectively, identified.

To understand the underlying mechanisms involved in Ba treatment, the iTRAQ protein database was analyzed by ingenuity pathway analysis (IPA) with reference to the Humans (Homo sapiens) database. The analysis was conducted with the filter set at *p* < 0.05. Upstream regulators and ingenuity canonical pathways were predicted by IPA. A total of 190 upstream regulators were found after Ba treatment (*p* < 0.05) with 18 regulators predicted with z-scores, which indicate a predicted activation or inhibition of the specific candidates (Table 2). Of these, vascular endothelial growth factor A (VEGFA) and basic fibroblast growth factor (FGF2) were found to have a significant activation z-score of 2 (activated) and −2 (inhibited), respectively. 

## 3. Discussion

Our results demonstrated that Ba (1) elicited a ~5-mmHg decrease in IOP along with a 90% increase in conventional outflow facility in mouse eyes; (2) inhibited cell volume regulation; and (3) triggered cell relaxation via downregulation of pMLC in primary hTM cells. Our proteomic data using iTRAQ also revealed changes in ECM-related proteins, potentially influencing the ECM remodeling and thereby contributing to a decreased outflow resistance.

Elevated IOP is a major and the only current modifiable risk factor for glaucoma. Thus, IOP-lowering therapy continues to be the first-line treatment to delay its onset and progression. Elevated IOP is caused primarily by impeded fluid drainage through the TM. Other than ROCK inhibitors, most anti-glaucoma medications act by either reducing AH secretion or facilitating AH drainage via uveoscleral outflow [21]. Research on agents facilitating fluid drainage through the conventional TM outflow pathway represents an appealing approach as TM is the diseased tissue responsible for increased IOP [22,23]. The current study ailed to explore a natural compound with potent action and few unwanted side effects. Ba is a natural flavonoid and its therapeutic potential has been associated with its anti-inflammatory, anti-oxidative, and anti-apoptotic properties [13,24]. Our results showed that Ba had no effects on hTM cell viability and proliferation in the micromolar range. These findings are consistent with published work in other cell types [25,26], suggesting that Ba has minimal cytotoxicity and is safe to use. Ba has been shown to protect neurons from ischemic and oxidative damage in various models of neuropathy [27,28,29], indicating its potential significance for glaucoma therapy. Previous studies have demonstrated that the regulation of TM cell volume, contractility, ECM synthesis, and remodeling all contribute to the maintenance of outflow resistance [7]. As Ba is found to regulate cell contractility [30,31], ion and fluid secretion [17,32], and ECM remodeling [33,34] in different cell types, suggesting that Ba may play a crucial role in modulating conventional outflow facility and IOP.

### 3.1. Ba Reduces IOP and Outflow Resistance through Modulating TM Cell Functions 

We showed that Ba induced an acute concentration-dependent increase in conventional outflow facility. At 10 µM, the outflow facility was increased by almost 90% and the increase in outflow facility was similar to our recently published results with 50 µM Y39983 (83% increase), a selective ROCK inhibitor [35]. The magnitude of Ba-induced increase in outflow facility was also comparable to other previous reports with ROCK inhibitors in different animal species [36,37]. These results strongly indicate that Ba is as effective as clinically-available anti-glaucoma drugs in enhancing conventional AH drainage. More importantly, Ba, when applied topically and intravitreally, resulted in a sustained IOP reduction, strongly supporting that Ba has a potential biomedical significance in glaucoma treatment by lowering IOP.

Outflow resistance in TM cells can be modulated by cell volume regulation [38,39,40]. Various ion transporters and channels have been identified in the TM cells responsible for volume regulation [41,42,43,44]. It is likely that fluctuation of AH’s osmolarity may trigger swelling- and stretch-activated channels in TM cells, leading to an alteration of outflow facility [44,45,46]. No changes in cell volume were observed by Ba at any of the concentrations tested in the isotonic bathing solution. In contrast, Ba triggered a concentration-dependent inhibition of RVD upon hypotonic stimulation. Generally, alteration of cell volume regulation triggers acute changes in outflow resistance [42,47,48]. It has been found that bumetanide (a blocker of Na^+^-K^+^-2Cl^−^), NS1619 (a BK_Ca_ activator), and bathing Cl^−^ substitution trigger a shrinkage of TM cells and increase outflow facility under isosmotic conditions [39,41]. Nitric oxide (NO) donor diethylenetriamine has also been reported to reduce TM cell volume and increase outflow facility [48]. In this study, Ba had no effect on cell volume under isosmotic conditions, indicating that Ba did not influence baseline steady-state cell volume homeostasis. However, Ba did inhibit the RVD upon hypotonic exposure. Our data are consistent with a recent study in which inhibition of anoctamin-6 was shown to modulate swelling-activated Cl^−^ current and RVD in TM cells, thereby reducing outflow resistance [49]. It has been reported that ATP release increases in TM cells following hypotonic cell swelling [50]. The ATP release stimulated by cell swelling is subjected to purinergic regulation [51] and alters ecto-enzymatic delivery of adenosine to A1ARs, which in turn modulates cytoskeletal remodeling through MMP secretion [52], facilitating AH drainage and IOP reduction [53,54]. 

In addition to volume regulation, we hypothesized that Ba regulates the contractility of TM cells. We observed that Ba mediated a concentration-dependent hTM cell relaxation. Contractility of TM cells can be regulated by agents including endothelin-1 [18,19], NO donor [18], and ROCK inhibitors [19,55,56], via MLC phosphorylation [18]. In agreement with this, Ba reduced the expression of pMLC in hTM cells. Whether or not the relaxation effect of Ba may be related to its inhibitory activity on ROCK and protein kinase C (PKC), as demonstrated in other cell types [25,30], awaits further investigation. Our results indicate that Ba modulates hTM cell contractility and volume regulation, potentially contributing to ECM remodeling and an increased outflow facility. 

### 3.2. Potential Protein Candidates Regulating Conventional Outflow Facility 

To better delineate the functional significance of Ba, a proteomic approach was employed to study the protein expression profile after Ba treatment in hTM cells. The rationale behind studying 3-h post-treatment was to identify early protein changes responsible for the acute effects. Ba (10 µM) was used because it was the lowest concentration shown to elicit changes in hTM cells, leading to a significant increase in outflow facility. Our results revealed 47 differentially-expressed proteins after Ba treatment and some of which were related to cytoskeleton rearrangement and ECM remodeling. For example, upregulation of CTSB, which belongs to cysteine cathepsins under the C1 papain family of cysteine proteases, was observed. CTSB is involved in cell adhesion, cell motility, and ECM degradation [57]. It has been shown that upregulation of CTSB may facilitate ECM degradation via caveolae-mediated endocytosis [58]. Recently, it has been shown that CTSB expression is inhibited in the TM of glaucoma patients [59]. The CTSB-induced attenuation of ECM deposition and fibrosis in TM cells [60] further suggests that CTSB upregulation by Ba may contribute to ECM remodeling, leading to an increased outflow facility. 

In contrast, DDT and PBXIP1 were downregulated after Ba treatment. DDT is the homologue of macrophage migration inhibitory factor (MIF) and has been regarded as the MIF-like cytokine responsible for inflammatory response [61]. PBXIP is a co-repressor of pre-B-cell leukemia homeobox 1, which participates in cell migration, proliferation, growth, and differentiation [62,63]. It has been shown that PBXIP1 promotes cell migration in gastric [64], thyroid [65], and human alveolar basal epithelial cells [66], through the PI3K/AKT signaling pathway. In addition, actin-related protein 2/3 complex subunit 1A (ARPC1A; Q92747; *p* < 0.05) was reduced with a fold change of 0.65 (*p* < 0.05). Actin-related protein 2/3 complex plays a crucial role in actin cytoskeletal functions [67]. Although there is limited evidence on ARPC1A, a study has demonstrated that silencing of actin-related protein 2/3 complex subunit 5 significantly reduced cell motility in squamous cell carcinoma [68].

Increased expression and activities of MMPs have been linked with ECM degradation through an IOP homeostasis feedback mechanism [69]. We also observed a small increase in MMP-14 expression after Ba treatment (*p* < 0.05). Other MMPs were; however, not detected in our proteomic study. This could be due to the sensitivity of the iTRAQ-MS and the fact that only TM cells were used in the study, rendering it difficult to detect secreted proteins, including MMP-2 and MMP-9. MMP-14 has been found to be upregulated in TM cells under mechanical stress [70]. More recently, it has been shown that genipin, an agent which induces matrix cross-linking, reduces outflow facility in porcine and human anterior segments, possibly mediated by a reduction of MMP-14 [71]. Likewise, trabodenoson is reported to increase outflow facility through MMP-14 [72]. The activation of VEGFA and inhibition of FGF2 predicted from IPA were also consistent with the hypothesis of Ba-induced reduction of ECM secretion and outflow resistance [73]. Taken together, the upregulation of CTSB and MMP-14 along with the downregulation of PBXIP1, DDT, and ARPC1A may serve as early signals for ECM remodeling, accounting for the observed increase in outflow facility.

### 3.3. Other Properties of Ba in Potentially Regulating TM Cell Functions 

Oxidative stress is implicated in glaucoma pathogenesis because elevated reactive oxygen species (ROS) have been found in the TM, AH, and RGCs of glaucomatous eyes [74,75,76]. Based on the STRING analysis, eight proteins related to the oxidation–reduction process were differentially expressed after Ba treatment of hTM cells. For example, pyruvate dehydrogenase E1 component subunit beta (PDHB; P11177; *p* < 0.05) was increased by 1.61-fold by Ba. PDHB has been found to be a major subunit of pyruvate dehydrogenase complex (PDHC), which plays an important role in aerobic energy metabolism [77]. The covalent catalysis with thiamin diphosphate induced by PDHB is suggested to be the rate-limiting step in PDHC catalysis [78], and the upregulation of PDHB by Ba is expected to enhance mitochondrial activities. An increase in lipid peroxidation in the mitochondria, which is induced by ROS, has been reported in various neurodegenerative diseases including glaucoma [79]. Patients with POAG have been found to have lower levels of ATP production and mitochondrial potentials in the TM [80]. In neurodegenerative diseases, Ba has also been found to inhibit lipid peroxidation and increase cell viability in PC12 [81] and SH-SY5Y cells [82]. These findings are consistent with our results which showed that upregulation of PDHB by Ba can potentially reduce ROS-induced mitochondrial dysfunction in hTM cells.

Loss of TM cellularity has been observed in patients with POAG [83]. This loss of TM cells may be due to damage triggered by oxidation and inflammation, contributing to an increased outflow resistance due to reduced ECM degradation [10]. Our results showed an upregulation of proliferation-associated protein 2G4 (PA2G4; Q9UQ80; *p* < 0.05) after Ba treatment (Table 1). Reduced expression of PA2G4 has been observed in aging retina with glaucoma [84]. Using episcleral vein occlusion, a significant loss of RGCs and downregulation of PA2G4 expression have been shown in aged rats receiving cauterization when compared with controls [85]. Recently, the neuroprotective effects of Ba against retinal functional and morphological damages resulting from retinal ischemia reperfusion injury have also been demonstrated [86]. It was found that Ba alleviates the expression of pro-inflammatory cytokines including TNF-α, IL-6, and IL-1β in mouse microglia, supporting its biomedical significance for glaucoma treatment as elevated levels of TNF-α, IL-6, and IL-1β are observed in the TM of glaucoma patients [87]. Overall, our results indicate that the anti-oxidative, anti-apoptotic, and anti-inflammatory properties of Ba may be important for the maintenance of the normal functions of TM and IOP homeostasis. 

## 4. Materials and Methods

### 4.1. Non-Invasive IOP Measurements

Adult C57BL/6J mice (aged 2–4 months) were kept in cages with daily 12-h light/dark cycles with unlimited food and water. All animal experiments were performed in compliance with the Association for Research in Vision and Ophthalmology Statement for the Use of Animals in Ophthalmic and Vision Research. Rebound tonometer (TonoLab, iCare) was used for IOP measurements under awake conditions. At least three readings were taken and averaged for data analysis. All IOP measurements were conducted at the same time of the day to minimize diurnal variation. For topical drug administration, 20 µL of 10 mM Ba or vehicle (2-Hydroxypropyl-β-cyclodextrin) was applied topically in the treatment eye twice (separated by 10 min), while the contralateral eye of the same animal was left untreated and served as a control. For intravitreal injection, animals were anesthetized by intraperitoneal injection of a mixture of ketamine (80 mg/kg) and xylazine (16 mg/kg). After that, 2 µL of 10 µM Ba or vehicle was injected into the treatment eye, while the contralateral eye of the same animal was untreated as control. In both experiments, IOP was monitored at baseline, 3, 6, and 24 h after treatment.

### 4.2. Measurement of Outflow Facility in Mouse Eyes

Freshly enucleated eyes from C57BL/6J mice were used. The conventional outflow facility was determined by the constant-pressure perfusion system as previously described [88,89]. Briefly, enucleated mouse eyes were kept in a water chamber at 37 °C, before cannulation of the anterior chamber with a 33-gauge beveled NanoFil needle tip (World Precision Instruments, Sarasota, FL, USA). The needle was connected via a pressure transducer (Honeywell, Charlotte, NC, USA) to a reservoir containing Dulbecco’s PBS with 5.5 mM D-glucose (D-PBSG) in a Hamilton glass syringe and placed on a motorized syringe pump (Harvard Apparatus, Holliston, MA, USA) under control of a computerized program (LabVIEW Software; National Instruments, Austin, TX, USA). The eyes of each animal were continuously perfused for at least 30 min with D-PBSG containing Ba for one eye and vehicle for its fellow eye. The outflow facilities of both eyes were then determined by measuring flow rates at sequential pressure steps from 4 to 20 mmHg. The outflow facility was derived from linear regression analysis between flow rates and pressure applied. Ba-treated and vehicle-treated eyes of the same animal were compared.

### 4.3. Preparation of hTM Cells 

Primary hTM cells were obtained from the Department of Ophthalmology, Duke University School of Medicine. Cell strains from six human donors (aged from 3 months to 88 years old) that had been characterized previously [90,91] according to consensus recommendations [92] were used. Cells were incubated at 37 °C until confluence and then maintained in low glucose Dulbecco’s modified Eagle’s medium (DMEM) (Invitrogen, Carlsbad, CA, USA) with streptomycin (100 mg/mL), penicillin (100 units/mL), glutamine (0.29 mg/mL), and 1% fetal bovine serum (FBS) (Invitrogen) for at least a week before use.

#### 4.3.1. Measurement of Cell Viability and Proliferation

The cytotoxicity of Ba was evaluated by Trypan blue exclusion assay as reported previously [93]. hTM cells were incubated in serum-free DMEM overnight before treatment. Subsequently, Ba, vehicle, or PBS were added to the cells and incubated for 2 and 4 days. Cells were harvested with 0.25% trypsin and centrifuged, re-suspended in PBS, and mixed with 0.4% Trypan blue in a 1:1 ratio. A total of 300 unstained (viable) and stained (dead) cells were counted. The cell viability was determined by the (no. of unstained cells/no. of total cells) × 100%.

The proliferation of hTM cells was evaluated by a 3-(4,5-dimethylthiazol-2-yl)-2, 5-diphenyltetrazolium bromide (MTT) assay (Thermo Fisher Scientific, Waltham, MA, USA) according to the manufacturer’s instruction [94]. 5 × 10^3^ hTM cells per well were seeded in 96-well plates. Ba, vehicle, or PBS were added to the cells and incubated for up to 4 days. After that, 10 µL of 12 mM MTT in PBS was added and incubated at 37 °C for 4 h. DMSO (50 µL) was added and incubated in the dark for 10 min with gentle shaking. The absorbance at 570 nm was determined by an AO microplate reader (Azure Biosystems Inc, Dublin, OH, USA).

#### 4.3.2. Measurements of Real-Time Cell Volume Changes

The effects of Ba on cell volume were monitored by electronic cell sorting [52,95]. hTM cells were harvested and centrifuged at 1,500 rpm for 5 min after confluence. The cell pellets were re-suspended in 30 mL of isotonic solution with a 40 μm nylon cell strainer (Falcon, Corning, New York, NY, USA) and settled for 30 min. The isotonic bathing solution contained (in mM): 110 NaCl, 1.2 MgCl_2_, 4.7 KCl, 2.5 CaCl_2_·H_2_O, 1.2 KH_2_PO_4_, 30 NaHCO_3_, 15 HEPES, and 10 glucose (290–305 mOsmol/kg H_2_O, pH = 7.4). Real-time cell volume measurements were conducted with a Coulter Counter (Beckmann Coulter, Inc, Brea, CA, USA) with a 100 μm aperture tube. Cell volume was determined by the peak of distribution function throughout the experiment. During the equilibration period, hTM cell volume was monitored every 5 min for 30 min. Subsequently, cell volume was monitored for 30 min after the addition of Ba or vehicle to the isotonic bathing solution. The RVD was triggered by exposing the hTM cells to a hypotonic solution for 30 min. The hypotonic solution was prepared similar to that of the isotonic solution except that the NaCl concentration was reduced to 37 mM (150–160 mOsmol/kg H_2_O, pH = 7.4).

#### 4.3.3. Measurement of Cell Contractility and Phosphorylated Myosin Light Chain (pMLC) Expression

The collagen gel (1.5 mg/mL) was prepared by mixing 4.42 mg/mL collagen (BD Bioscience) with 10× PBS, 1 M NaOH and water [18,96]. Cells were seeded on the gel (1.5 × 10^5^ cell/cm^2^) in a 48-well plate at 37 °C for 24 h and then incubated in serum-free media overnight. The collagen gels were detached from the well using 200 µL MultiFlex round tips (Sorenson^TM^ BioScience, Inc) and settled at 37 °C for 9 h [18]. Ba or vehicle was added and incubated for 3 and 6 h. Carbachol (5 μM) was used as a control [19]. The gel area was monitored at baseline, 3 h, and 6 h after drug treatment. Measurements of gel areas were determined using the Motic analysis program (Moticam BTW 8, MMS Microscopes).

Phosphorylation of MLC was determined by measuring phosphorylated MLC (pMLC) as a percentage of total MLC expression using Western blot analysis [18,19,20]. Cells were treated with Ba or vehicle for 5 min. Cells were lysed in lysis buffer (7 M urea, 2 M thiourea, 30 mM TRIS, 2% CHAPS, and 1% ASB14, pH = 8.5) with phosphatase and protease inhibitors. Samples were incubated at 4 °C for 1 h with sonication. Total proteins were quantified using Bradford protein assay (Bio-Rad, Hercules, CA, USA). For this purpose, 30 µg proteins were mixed with β-mercaptoethanol and Laemmli Sample Buffer (BioRad), heated at 95 °C for 5 min, separated in 12% polyacrylamide gels, and transferred onto polyvinylidene fluoride (PVDF) membranes, which were incubated with primary (1:1000) antibodies at 4 °C overnight for detection of phosphor-specific MLC 2 (Thr19/Ser18) and MLC 2. After washing, the blots were incubated with secondary antibodies (1:5000) before visualization with Amersham ECL Select Western Blotting Detection Reagent (GE Healthcare - Life Sciences) and imaged by ChemiDoc^TM^ MP Imaging System (Bio-Rad). The changes in signal intensity of pMLC and MLC were determined by Image Studio Lite (version 5.2).

### 4.4. Proteomics Study of Ba in hTM Cells

iTRAQ proteomics was performed using an 8-plex isobaric tag (SCIEX, US) that allowed all Ba- and vehicle-treated samples to be processed and labeled in the same run for mass spectrometry.

After treatment with Ba or vehicle, cells were treated with lysis buffer (7 M urea, 2% CHAPS, 0.1 M TEAB, and protease inhibitor). The lysate was then sonicated at 4 °C for 30 min and centrifuged for 15 min at 4 °C. Subsequently, the supernatant was collected and proteins purified by pre-cooled 100% acetone at a 1:4 ratio at −20 °C overnight. After centrifugation, at 15,000 rpm for 30 min at 4 °C, 500 µL, 80% acetone was added to the pellets for washing, before a further 20 min centrifugation at 4 °C. After collecting the sample pellets, proteins were reduced and blocked by cysteine according to the (Sciex, US) manufacturer’s suggested protocol. 

The pellets were re-suspended in 10 µL 7 M urea in 0.5 M TEAB and protein concentrations were measured by Bradford protein assay (Bio-Rad). Then, 50 µg of protein was collected from each sample. TCEP, as a reducing agent, was added and incubated for 1 h followed by cysteine blocking at room temperature for 10 min. 0.1 M TEAB was added to obtain a final concentration of 1 M urea and samples were incubated at 37 °C with trypsin (1:20 trypsin to total protein) for 16 h with gentle agitation.

Before labeling, ZipTip (ZipTip C18, Millipore, Burlington, MA, USA) was used to clean up the samples. The final peptide concentration was measured by peptide assay (PieceTM Quantitative Colormetric Peptide Assay, Thermo Scientific, Waltham, MA, USA). Then, the sample digests were dried at 4 °C by speed-vac. 12.5 µL TEAB (0.5 M) was added to dissolve the peptides. Each digest was labeled with 25 µL of iTRAQ^®^ Reagent-8-plex for 2 h at room temperature and then mixed with 50 µL isopropanol. After labeling each sample, the contents of each iTRAQ^®^ Reagent-8plex-labeled samples were combined and mixed.

Mass spectrometry acquisition was conducted using a reverse phase high pressure liquid chromatography electrospray ionization tandem mass spectrometry (RP-HPLC-ESI-MS/MS) TripleTOF^®^ 6600 mass spectrometer (AB SCIEX) with Analyst TF 1.7 software. For DDA, iTRAQ labeled peptides (2 µg) were loaded onto a trap column (350 µm × 0.5 mm, C18) by loading buffer (0.1% FA, 2% acetonitrile in water) at 2 µL/min for 15 min. Proteins were then separated on a nano-LC column (100 µm × 30 cm, Smartube C18, 5 µm) using an Ekisgent 415 nano-LC system.

For MS data analysis, data-dependent acquisition was searched against Homo sapiens Uniprot reviewed database (version, 26,095 entries). Protein identification (ID) was performed using ProteinPilot 5.0 software (SCIEX). iTRAQ-8-plex (peptide labeled) was selected as the sample type and trypsin as the enzyme. Other parameters selected included: cysteine alkylation using MMTS, thorough search effort, biological modification, quantitate, bias correction, and background correction. A 1% FDR was set as the filter for protein identification. Only proteins containing at least two peptides were analyzed for quantification.

The Protein ANalysis THrough Evolutionary Relationships (PANTHER) classification system (version 15 released Feb, 2020, analysis performed in March 2020) Geneontology Unifying Biology was used for the protein classification for all significantly regulated proteins (*p* < 0.05) based on gene ontology analysis (homo sapiens). The imported proteins were classified in terms of molecular functions, protein classes, cellular component-cellular anatomical entity, and biological processes. For upstream regulator and ingenuity canonical pathways analysis, ingenuity pathway analysis (IPA, Ingenuity Systems, Redwood City, CA, USA) software was used. Only iTRAQ protein database with at least two unique peptides were imported for analysis. The species was set to human and a *p*-value of <0.05 (paired *t*-test) was adopted. The possible upstream regulator and ingenuity canonical pathways were predicted by the calculated significance using the Core Analysis in IPA. It was based on a comparison between the database of Ingenuity^®^ Knowledge Base and the input protein list. 

### 4.5. Data Analysis

All data were expressed as Mean ± SEM. SigmaPlot for Windows (version 13.0) was used for statistical analysis. Student’s *t*-test, one-way, and two-way Analysis of Variance (ANOVA) followed by Bonferroni *t*-test were used. A *p*-value of < 0.05 was considered statistically significant (* *p* < 0.05; ** *p* < 0.01; *** *p* < 0.001).

## 5. Conclusions

The current study results reveal that Ba acts by modulating, at least in part, TM cell relaxation and volume regulation, potentially leading to ECM remodeling and influencing the outflow facility and thereby IOP. Loss of TM cells has been reported in glaucoma patients, resulting in reduced ECM degradation and an impeded AH drainage. The anti-oxidative, anti-inflammatory, and anti-apoptotic properties of Ba also provide new insight into developing novel and disease-modifying targets for strengthening and restoring the normal TM cell functions, eventually improving AH drainage and IOP homeostasis. 

## Figures and Tables

**Table 1:** Quantitative differential protein expression with ≥1.3-fold change after Ba treatment (17 upregulated and 14 downregulated proteins). (*n* = 3, * *p* < 0.05; ** *p* < 0.01; *** *p* < 0.001, paired *t*-test).

Gene Name	Protein Name	Accession No	Fold Change	Log_2_ (Fold Change)	*p*-Value
NAGA	Alpha-N-acetylgalactosaminidase	P17050	0.2	−2.3	**
PBXIP1	Pre-B-cell leukemia transcription factor-interacting protein 1	Q96AQ6	0.4	−1.3	*
SAE1	Isoform 3 of SUMO-activating enzyme subunit 1	Q9UBE0-3	0.4	−1.2	**
BCAP29	Isoform 2 of B-cell receptor-associated protein 29	Q9UHQ4-2	0.5	−1.2	*
AKR1B10	Aldo-keto reductase family 1 member B10	O60218	0.5	−1.2	*
PSMD10	26S proteasome non-ATPase regulatory subunit 10	O75832	0.5	−1.1	**
PDCD6	Programmed cell death protein 6	O75340	0.5	−1.1	*
XRCC6	X-ray repair cross-complementing protein 6	P12956	0.6	−0.8	*
DDT	D-dopachrome decarboxylase	P30046	0.6	−0.8	***
MANF	Mesencephalic astrocyte-derived neurotrophic factor	P55145	0.6	−0.8	*
ARPC1A	Actin-related protein 2/3 complex subunit 1A	Q92747	0.7	−0.6	*
MIF	Macrophage migration inhibitory factor	P14174	0.7	−0.6	*
KLC1	Isoform I of Kinesin light chain 1	Q07866-9	0.7	−0.5	*
DAB2	Disabled homolog 2	P98082	0.7	−0.5	*
AHCYL1	S-adenosylhomocysteine hydrolase-like protein 1	O43865	1.3	0.3	*
TUFM	Elongation factor Tu, mitochondrial	P49411	1.3	0.3	*
ATP5PO	ATP synthase subunit O, mitochondrial	P48047	1.3	0.4	*
EML4	Echinoderm microtubule-associated protein-like 4	Q9HC35	1.3	0.4	*
RUVBL2	RuvB-like 2	Q9Y230	1.3	0.4	*
RBMX	RNA-binding motif protein, X chromosome	P38159	1.4	0.4	*
ACTA2	Actin, aortic smooth muscle	P62736	1.4	0.5	**
CTSB	Cathepsin B	P07858	1.5	0.6	**
PA2G4	Proliferation-associated protein 2G4	Q9UQ80	1.5	0.6	*
CARS	Isoform 3 of Cysteine--tRNA ligase, cytoplasmic	P49589-3	1.5	0.6	*
PDHB	Pyruvate dehydrogenase E1 component subunit beta, mitochondrial	P11177	1.6	0.7	*
SLC25A3	Isoform B of Phosphate carrier protein, mitochondrial	Q00325-2	1.8	0.8	*
XPNPEP1	Xaa-Pro aminopeptidase 1	Q9NQW7	2.2	1.1	*
TLR7	Toll-like receptor 7	Q9NYK1	2.2	1.2	*
PFDN6	Prefoldin subunit 6	O15212	2.7	1.4	*
CPA4	Carboxypeptidase A4	Q9UI42	3.1	1.7	**
MGARP	Protein MGARP	Q8TDB4	3.8	1.9	*

**Table 2:** Upstream regulators predicted from the IPA after 3 h Ba treatment (*p* < 0.05). Calculated z-score reflects the overall predicted activation state of the regulator (negative value: inhibition, positive value: activation). Values of |z|>2 are considered significant. (* *p* < 0.05; ** *p* < 0.01; *** *p* < 0.001).

Upstream Regulator	Description	Molecule Type	*p*-Value	Activation z-Score
TP53	Tumor protein p53	transcription regulator	**	0.7
NFKBIA	NF-kappa-B inhibitor alpha	transcription regulator	**	0.8
MAPK14	Mitogen-activated protein kinase 14	kinase	**	1.2
GATA6	GATA binding protein 6	transcription regulator	**	−1.1
CDKN1A	Cyclin dependent kinase inhibitor 1A	kinase	**	0.5
OGA	O-GlcNAcase	enzyme	**	1.3
HIF1A	hypoxia inducible factor 1 subunit alpha	transcription regulator	**	0.2
AGT	Angiotensinogen	growth factor	**	−0.4
TGFB1	transforming growth factor beta 1	growth factor	**	0.6
AKT1	AKT serine/threonine kinase 1	kinase	**	0.2
MYC	MYC Proto-Oncogene, BHLH Transcription Factor	transcription regulator	**	−1.6
VEGFA	vascular endothelial growth factor A	growth factor	*	2
MAPK1	mitogen-activated protein kinase 1	kinase	**	0.2
PTEN	phosphatase and tensin homolog	phosphatase	**	0.4
EGFR	epidermal growth factor receptor	kinase	**	0.6
FGF2	fibroblast growth factor 2	growth factor	**	−2
CEBPB	CCAAT Enhancer Binding Protein Beta	transcription regulator	**	−0.8
IL1B	interleukin 1 beta	cytokine	**	−0.9

## Data Availability

The data presented in this study are available in request from the corresponding author.
